# Religion and the public ethics of stem-cell research: Attitudes in Europe, Canada and the United States

**DOI:** 10.1371/journal.pone.0176274

**Published:** 2017-04-20

**Authors:** Nick Allum, Agnes Allansdottir, George Gaskell, Jürgen Hampel, Jonathan Jackson, Andreea Moldovan, Susanna Priest, Sally Stares, Paul Stoneman

**Affiliations:** 1 Department of Sociology, University of Essex, Colchester, England; 2 Fondazione Toscana Life Sciences, Siena, Italy; 3 Department of Methodology, London School of Economics, London, England; 4 Department of Sociology of Technology and Environment, University of Stuttgart, Stuttgart, Germany; 5 Independent Consultant, Communication for Science, Energy, and Environment, Seattle, United States of America; 6 Department of Sociology, City University, London, England; 7 Department of Sociology, Goldsmiths College, London, England; Johns Hopkins School of Medicine, UNITED STATES

## Abstract

We examine international public opinion towards stem-cell research during the period when the issue was at its most contentious. We draw upon representative sample surveys in Europe and North America, fielded in 2005 and find that the majority of people in Europe, Canada and the United States supported stem-cell research, providing it was tightly regulated, but that there were key differences between the geographical regions in the relative importance of different types of ethical position. In the U.S., moral acceptability was more influential as a driver of support for stem-cell research; in Europe the perceived benefit to society carried more weight; and in Canada the two were almost equally important. We also find that public opinion on stem-cell research was more strongly associated with religious convictions in the U.S. than in Canada and Europe, although many strongly religious citizens in all regions approved of stem-cell research. We conclude that if anything public opinion or ‘public ethics’ are likely to play an increasingly important role in framing policy and regulatory regimes for sensitive technologies in the future.

## Introduction

A number of development trajectories in the domain of modern biotechnology–most notably the fate of GM food in Europe–have demonstrated the centrality of public concerns in sustainable technology development. The reception of new technologies by the public is linked to judgements about risks and benefits, but it is also based on ethical issues and general ideas about ‘how we want to live,’ and this is particularly the case for sensitive technologies in the life sciences. Given the ongoing explosion of new gene-based technologies such as synthetic biology, cloning, gene editing and personalized medicine, we seek in this paper to add historical context to such debates by examining an exemplar case–that of human embryonic stem-cell research.

The history of political and public debates about novel interventions in the process of reproduction appears to repeat itself. Recent controversies over the future of stem-cell research [1, 2] echo elements of earlier debates on contraception and in-vitro fertilisation (IVF), but they also prefigure more recent ones on synthetic biology and gene editing [3, 4]. At the centre of these debates is the question: ‘what are the limits to human intervention in matters of life?’ Offering the opportunity of sex without reproduction, contraception was considered by some to be an aberration and a threat to the moral order. Much the same was said of IVF, which brought the possibility of reproduction without sex. Yet with some exceptions both contraception and IVF are now broadly accepted and in widespread use.

Two decades have passed since ‘Dolly’ the cloned sheep swept the world’s headlines, generating moral outrage over the boundaries of acceptable interference in creation [5]. Research has continued and the debate has become more nuanced, most notably after the isolation of embryonic stem-cells; and we saw the distinction between therapeutic and reproductive cloning being developed in public discourse [6]. As research evolved into the new millennium, stem-cell research became one of the most contested issues in science policy making. While stem-cell research is heralded as a major breakthrough in biomedical science, as Nielsen notes, it was ‘attained with cloning having the dubious status of the most promising as well as the most controversial among the many emerging biotechnologies’ [7]. The National Research Council in the U.S. wrote that stem-cell research has led ‘scientists and non-scientists alike to contemplate profound issues, such as who we are and what makes us human beings.’ [8].

### Public ethics

Alongside elite discourse and debate, amongst scientists, legislators, regulators and ethicists, the opinions of lay publics have been, and continue to be, important. We use the term ‘public ethics’ not because we assume that public opinions are necessarily evidence of intensive deliberation or elaboration by the public such as that practiced by professional ethicists. Rather it is because we regard it as plausible to map some important attitudinal dimensions onto established ethical principles or moral positions. The reason for this comes from recent thinking in empirical moral psychology. In this field, recognized ethical and moral positions are regarded as formalisations of existing more fundamental attitudes and intuitions, rather than the latter being led by the former [9]. This being the case, such public ethics or opinions can act to constrain or enable scientific and technological development in democratic societies [10]. Where such developments tap into especially ‘hot button’ issues or themes, public attitudes can, and do, come to the attention of politicians and regulators and sometimes have palpable effects on policy regimes and funding priorities. For example, the unofficial EU moratorium on commercial planting of genetically modified (GM) crops was one of the higher profile instances of politics and science colliding in the form of a World Trade Organisation (WTO) dispute. European public opinion was–and remains–negative and very few GM crops are farmed in Europe despite a scientific consensus for their safety. Underlining this, one of the key biotech firms, BASF, moved its research operations to the U.S. in 2012, citing consumer resistance [11, 12]. That the controversy over GM crops led to a trade dispute between the U.S. and the EU underscores the importance of a comparative approach to understanding the foundations for public opinion about contentious science and technology. To cite another contemporary example, within the US, public beliefs about anthropocentric global warming split along partisan lines [13] and the policy positions of Democrats and Republicans have to be understood in the context of the opinions of their supporters. Different cultural sensitivities mean therefore that it cannot be assumed that public opinion towards new scientific and technological developments will align in the same way in different parts of the world or in different socio-political contexts.

The first step for the current paper is to investigate and map out cross-national differences in public opinion on stem-cell research. The second is to examine what may underlie such differences as are observed, such that once such common factors are accounted for these cross-national differences are attenuated or eliminated. Drawing on data from three comparative social surveys, which together captured public opinion in Western Europe, the U.S. and Canada at a time when the stem-cell debate was at its height (in the mid-2000s), we investigate factors underlying divergences in public perceptions. Of particular interest in the current research is the extent to which particular attitudes underlying approval of stem-cell research assume varying importance in different parts of the world.

### Stem-cell research and ethical dilemmas for policy makers

The controversies surrounding human embryonic stem-cell research bring into focus two ideal-type ethical positions or world-views, which we could refer to as a ‘sanctity of life’ ethic and a ‘quality of life’ ethic. The ‘sanctity of life’ ethic outlines a deontological position that sees the embryo as a human being that, as such, possesses rights that are inviolable. Given that stem-cell research involves the destruction of embryos, it would be unethical to pursue it [14]. By contrast, a ‘quality of life’ ethic argues for a utilitarian approach: a paramount duty to alleviate suffering, and given that this research may lead to cures for some serious diseases, it would be unethical *not* to pursue it.

A starting premise of our study is that, when considering the viability of stem-cell research, researchers, politicians, regulators and the public at large are confronted by a classic dilemma: namely, how to come to terms with these two competing perspectives. Across Europe, the United States and Canada, politicians and regulators have adopted different resolutions to this dilemma. An adoption of a ‘sanctity of life’ position was initially evident in the U.S., where the Bush administration limited federally funded stem-cell research to the use of a small number of existing cell lines. Congressional bills to allow the federal government to fund embryonic stem-cell research using supernumerary embryos from fertility clinics were vetoed by President Bush in 2006 and 2007. The premise of this position was

‘…*not* an attempt to answer the question of how the government might best advance embryonic stem-cell research while conforming to the law on the subject. Rather, it [was] an attempt to answer the question of how the government might avoid encouraging the (presumptively) unethical act of embryo destruction and still advance the worthy cause of medical research.’ [15]

Some governments across the EU likewise issued similar cautious edicts based on concern for the sanctity of life vested in human embryos. In November 2005 an ethical declaration was signed by Austria, Germany, Italy, Luxembourg, Malta, Poland and Slovakia that called for the exclusion of any kind of stem-cell research from European public funding under the seventh framework programme for research that ran from 2007 to 2013.

In the U.S., the policy position adopted by the Bush administration was reversed in 2009 when an Executive Order was issued by President Barack Obama:

‘For the past 8 years, the authority of the Department of Health and Human Services, including the National Institutes of Health (NIH), to fund and conduct human embryonic stem-cell research has been limited by Presidential actions. The purpose of this order is to remove these limitations on scientific inquiry, to expand NIH support for the exploration of human stem-cell research, and in so doing to enhance the contribution of America's scientists to important new discoveries and new therapies for the benefit of humankind.’ [16]

This lifting of restrictions on federal funding of stem-cell research arguably now placed the U.S. alongside ‘quality of life’ proponents found in some EU member states. In Denmark, Greece, Finland, France, the Netherlands and Spain a liberal form of regulation allows for the procurement of stem-cell research from supernumerary embryos. Belgium, Sweden and the UK took a step further, legalising the creation of human embryos for the procurement of embryonic stem-cells, but only under strict conditions.

Despite some national differences within Europe on the issue of stem-cell research, notably in Germany and Portugal [17], the European Union adopted a compromise position, albeit leaning towards a regime where research could take place. In June 2006 the EU Parliament approved the Commission’s proposal to support the use of public funds for stem-cell research. Subsequently, the European Council of Ministers agreed to allow the use of public funds for stem-cell research, and only to prohibit the use of public funds for the procurement of new embryonic stem-cell lines. This echoes the policy climate in Canada, which legalised stem-cell research in 2004 on embryos that are surplus from fertility clinics, but retained a ban on therapeutic cloning and the creation of embryos for research.

Across the three regions we can see, then, three distinct policy climates, ranging from a negative policy of restriction in the U.S. under the Bush administration, to a compromise position in Canada, to a position of broadly positive engagement in Europe. It has been argued that this kind of variation in regulatory regimes exists partly due to the strong influence of religion on politics in the U.S. in relation to Western Europe and to a lesser extent Canada [18]. This view perhaps overstates the causal influence of religion on political attitudes, but it is nevertheless true that conservative ideologies tend at least to be associated closely with religious culture in the U.S. and are often expressed more in 'moral' imperatives rather than the more liberal concerns of equality and collective utility [19]. This religio-cultural climate is often contrasted with the more secular-based style of government in Western Europe, particularly when considering the influence of prevailing religious norms on social and political attitudes [20].

## The present research

Since embryonic stem-cell research came to public attention in the early 2000s, the broad trend for public perceptions has been for either stability or a relaxing of concerns. These trends have also led towards greater convergence in public opinion internationally, although the bases on which public perceptions are founded may be quite different. Understanding these bases is the focus of the present enquiry. According to the Virginia Commonwealth University Life Science Survey series (a nationally representative random-digit-dial survey), public support for stem-cell research in the U.S. rose from 40 percent in 2002 to around 65 percent in 2010 [21]. Another survey, carried out in 2005 by Knowledge Networks on behalf of the Genetics & Public Policy Center, found that 67 percent of Americans approved of stem-cell research. Interestingly, while overall approval was high, the same survey found that all but 12 percent of respondents held at least some conflicting views about the need to preserve embryos versus the need to pursue research [22]. That attitudes were far from unambiguous illustrates the need to look beyond simple approval ratings in order to understand public views on stem-cell research.

When it was last systematically measured with Eurobarometer surveys [23, 24], the percentage of Europeans approving of embryonic stem-cell research with either usual, or tighter regulations, was around 65 percent in both 2005 and in 2010. In Canada, the most recent evidence comes from an Angus Reid survey, which estimated the moral acceptability of stem-cell research was espoused by 65 percent of adult Canadians [25].

While we have some evidence that attitudes between our three regions have converged in recent times, our interest in the current study is in understanding what the differing bases of these attitudes were at a time when debate was at its height and most salient in the minds of the public, in the mid-2000s. The variation between the U.S., Canada and Europe in styles of governance, regulatory regimes and cultural contexts provides a useful comparative framework to explore differences in attitudes towards stem-cell research. One of the hypotheses that we assess in what follows is that national differences in approval of stem-cell research may be explained by different levels of religiosity. Furthermore, we would expect to see religiosity as being associated with more negative attitudes towards stem-cell research. Moreover, we would expect to see this relationship having its strongest effect in the U.S., then Canada and finally Europe.

Going beyond religiosity itself, the foregoing suggests that the ethical perspectives associated with religious versus secular based governance might also be reflected in the roots of public attitudes in these different regions. We would expect, therefore, that moral concerns were more influential for Americans and Canadians than for Western Europeans in coming to a judgment about stem-cell research. Concomitantly, we would also expect that considerations of benefits more than moral concerns have played a greater role for public attitudes in Western Europe than across the Atlantic. To summarise then, our research questions, which we seek to answer with data gathered during this critical period, are as follows:

- What is the difference between the U.S., Canada and Western Europe in levels of public approval of stem-cell research?
- What is the influence of each of two types of perspectives on approval–moral concerns and perceptions of benefits?
- How do these influences vary in their importance across the U.S., Canada and Western Europe?

To answer these questions, we tested a series of regression models to demonstrate firstly the extent to which attitudes differ between the U.S., Canada and Western Europe, after accounting for differences in the demographic composition of the three regions. Secondly we assessed the role of ethical considerations using two variables that broadly capture moral and benefit-based (or utilitarian) concerns. Finally, we investigated the relative importance of these perspectives across the three regions.

## Data and methods

### The surveys

Eurobarometer 64.3 on Biotechnology was the sixth in the series of surveys of public perceptions of biotechnology that began in 1991. The survey was fielded in 2005 in 25 member states of the European Union and afforded a unique opportunity to carry out comparative research between Europe and North America, with a set of harmonised questions being asked in each region. Multi-stage random sampling procedures were used to provide a statistically representative sample of national residents aged 15 and over, with a total sample of circa 25,000 respondents interviewed face to face [26]. The Canadian Biotechnology Strategy Secretariat's ‘International Public Opinion Research on Emerging Technologies’ survey was conducted during January and February 2005 in Canada and in the U.S.. Random digit dialling was used to select representative probability samples of 2,000 respondents in Canada and 1,200 respondents in the U.S., all aged 18 years or above, who were interviewed by telephone [27, 28]. Both the the Canadian and European surveys had a split-ballot design where only a random half of the respondents were asked all of the questions included in the analyses presented here.

### The response variable: Support for stem-cell research

Canadian and U.S. respondents were given the following description:

‘stem-cell research involves the use of special human cells to study diseases and their cures. stem-cells have the unique ability to grow into any type of cell in the human body. stem-cell research has led to breakthroughs in our understanding of diabetes, MS, and Parkinson’s disease that offer the potential for new treatments and cures. However, to conduct this research, scientists have to get stem-cells. They have been getting them from human embryos that are less than 2 weeks old and have been frozen and stored in fertility clinics. However, these embryos will only be used for research if they are not going to be used for fertility treatments. A recently discovered way of getting them is to extract stem-cells from the blood contained in umbilical cords that people could donate to research after giving birth. The umbilical cords would in most cases be frozen and stored for future scientific use’.

Following this description, respondents in U.S. and Canada were asked: ‘Overall, which of the following best captures your views about stem-cell research?’. European respondents were given a shorter but similar description:

‘stem-cell research involves taking human cells either from human embryos that are less than 2 weeks and that will never be transplanted into a women body old or from the blood in umbilical cords to grow new cells which can be used to treat certain diseases in different parts of the body’.

The question posed alongside this description is very similar to the U.S. and Canadian version: ‘Overall, which of the following best captures your views about using stem-cells?’ However, in Europe, respondents were asked separately for their views on research using embryonic stem-cells, and research using non-embryonic stem-cells. For the latter, they were presented with the following qualified description: ‘Suppose scientists were able to get all the stem-cells they need for research from umbilical cords and no longer had to get them from embryos’. While the two formulations of the context in which stem-cell research is to be approved or not are somewhat different, we do not have serious cause to believe that question wording differences greatly affect the comparability.

The response alternatives for these questions in both surveys were as follows:

I approve the use of stem-cell research, as long as the usual levels of government regulation and control are in place;

I approve of stem-cell research if it is more tightly controlled and regulated;

I do not approve of stem-cell research except under very special circumstances; and,

I do not approve of stem-cell research under any circumstances.

Bearing in mind variation in national regulatory frameworks for stem-cell research, it could be argued that the response ‘I approve the use of stem-cell research, as long as the usual regulations apply’ indicates markedly different levels of support in different countries. However, drawing on Sudman et al [29], we conjecture that respondents use the response alternatives as important cues in the interpretation of the meaning of the question. As such, they would consistently interpret the four alternatives as ordered categorical points representing a continuum from approval through increasing strictness of regulation to a veto. This means that, for example, even in those contexts in which stem-cell research is not permitted by regulation, respondents who are opposed to stem-cell research would select alternative 4 rather than 1. For the descriptive statistics, we dichotomised this variable to distinguish approval from disapproval, while in the regression models, we use the full scale.

### Measuring religious commitment

We use the reported frequency of attendance at religious services as a proxy for religious commitment. Respondents in Europe were asked: ‘Apart from weddings or funerals, about how often do you attend religious services?’ Respondents in the U.S. and Canada were asked: ‘In the past, how often have you attended a service at a place of worship?’, and interviewers were given the instruction: ‘If asked, do not include weddings and funerals.’ In both surveys the response alternatives were: ‘More than once a week’; ‘At least once a week’; ‘Several times a month’; ‘At least once a month’; ‘A couple of times a year’; ‘About once a year’; and ‘Never.’

Using the reported frequency of attendance at religious services as a proxy for religious commitment clearly oversimplifies a set of complex issues regarding the nature of religious faith and practice. Nevertheless, the measure of religiosity that we employed here is in widespread use in political science and sociology (for instance, it is repeated annually in the General Social Survey [30]) and has been shown to correlate strongly with other measures of religious identity as well as political and social attitudes [31, 32]. Conceptually and empirically social scientists often distinguish between religiosity, membership and practices. A propos of this it may be asked, what of Muslims who may, simply as a matter of course, attend services more frequently than Christians? What of those with a faith who do not attend religious services?

Our justification for the simple measure of frequency of attendance is based on pragmatic grounds. In Europe, Canada and the U.S., Christianity (combining Protestants, Catholics and the Orthodox) is the dominant religion–over 70%. The Christian churches have reasonably similar patterns of services, thus it may be expected that, by and large, the frequency of church attendance is related to religious commitment. After the Christians the next largest group is atheists/agnostics at 10–15% with Muslims and Jews between 1 and 2 percent [26]. Thus, in a national sample survey of 1,000 persons (as in the Eurobarometer) we would expect between 10 and 15 Muslims. As such, without much larger and costlier samples, or booster samples for certain religious groups, it is simply not worth collecting detailed information on religion, because any analysis could not be generalised with confidence. That said, a comparison between those of the Catholic and Protestant churches might have been of interest. Protestants are the largest group in the U.S. (although there are many varieties) while Catholics are the largest in Europe [33]. However, this question of denomination was not asked in the U.S. and Canadian surveys.

### Measuring moral acceptability and perceived benefits of stem-cell research

In the U.S. and Canada, respondents were asked:

‘In terms of the moral or ethical aspect of this research, again using the 1–5 scale, where 1 means that stem-cell research is morally unacceptable, 5 means it is morally acceptable, and the mid point 3 means it is morally questionable, how do you view this kind of research?’

They were also asked:

‘I would like to understand the extent to which you think stem-cell research might benefit our society. Using a scale of 1–5, where 1 is no benefit and 5 is substantial benefit, and the mid-point 3 is moderate benefit, how beneficial do you think stem-cell research will be to our society?’

In Europe, respondents were asked whether they agreed or disagreed to the following two statements:

‘It is ethically wrong to use human embryos in medical research even if it might offer promising new treatments’;

‘stem-cell research will help with cures and treatments for serious diseases’.

The response alternatives were: ‘Totally agree’; ‘Tend to agree’; ‘Tend to disagree’; and ‘Totally disagree’. To achieve comparability between the two surveys, the data from the U.S. and Canada were rescaled to conform to the range of 1 to 4 to match the European question. This facilitated easier comparison of effect sizes in the regression models. For the purposes of showing descriptive statistics, to enhance the clarity of the tables, responses were recoded into a binary variable where the value of one indicates positive views (i.e. greater than 2 on the longer scaled version) and zero otherwise.

### Control variables

In addition to these substantive variables, we also included controls for age, education and gender. Age was captured with binary variables representing age groups 15–24, 25–34, 35–44, 45–54, 55–64, 65+. Education was measured with a set of binary variables denoting the following levels: less than high school, high school, some college or greater, still studying. We also include “don’t know” responses to this question, as there were a small but non-trivial number of these in the European sample (94). Gender was recorded as a binary indicator.

## Results

Levels of overall support for stem-cell research vary somewhat by region. Table 1 summarises these variations. Combining the approval scores and working across the table, we can see that Europeans are the least approving out of the three regions (62 percent), compared to the data from the U.S. (73 percent) and Canada (81 percent). The combined disapproval rates demonstrate slightly less variation—Europe (23 percent), U.S. (27 percent) and Canada (18 percent). The major difference between the regions, however, is that European respondents are far more likely to say “don’t know” (15 percent) compared to the U.S. and Canada (at most 1 percent). If we disregard “don’t know”s from the analysis then the proportions in each region expressing approval become more closely aligned.

**Table 1:** Attitudes towards stem-cell research in Europe, the United States and Canada.

*% respondents within region/country*	Europe			United States	Canada
	Embryonic	Non-embryonic	Mean		
Approve with usual levels of government regulation and control	23	28	25	41	36
Approve if more tightly controlled and regulated	36	37	37	32	45
Do not approve except under very special circumstances	17	14	15	19	14
Do not approve under any circumstances	9	7	8	8	4
Don't know	15	14	15	1	0
Base*	10,192	10,312		1,200	1,000

* Not all questions were asked of all respondents, hence the reduced sample sizes in Europe and the U.S.

Religious commitment–captured in terms of frequency of attending religious services–is typically higher in the U.S. than in Canada, and typically slightly higher in Canada than in Europe. In our data, 12 percent of Americans report attending religious services more than once a week, compared to 4 percent of Canadians and 3 percent of Europeans; by contrast, only 19 percent of American respondents attend less than once a year, compared to 31 percent of Canadians and fully 40 percent of Europeans.

From the outset, we expected that greater religious commitment would be associated with weaker support for stem-cell research. We also hypothesised that religiosity would be a more important basis for approval or disapproval of stem-cell research in the U.S. compared to Canada and Europe. Table 2 shows that in all regions, approval of stem-cell research does indeed decline as religious commitment increases. The pattern is less pronounced in Europe than in the U.S. and Canada, lending initial support for our hypothesis. It is important to note, though, that even among the most religious, around half of the public approves of stem-cell research in the U.S., Canada and in Europe.

**Table 2:** Attitudes towards stem-cell research by religious attendance.

	More than once a week	Once a week	About once a month	Two times a year or less
**U.S.**				
Approve	48	63	77	87
Disapprove	51	35	22	12
Don’t know	1	1	1	1
Total	100	100	100	100
**Canada**				
Approve	49	70	81	86
Disapprove	49	30	19	14
Don’t know	2	0	0	1
Total	100	100	100	100
**Europe**				
Approve	49	46	56	64
Disapprove	26	26	24	20
Don’t know	24	28	20	16
Total	100	100	100	100

These descriptive results suggest that religion is strongly associated with support for and opposition to stem-cell research in all regions. In the next section we present multivariate models where we have explored in more detail the extent to which, alongside religion, different ethical perspectives are associated with approval, and how this varies by region.

### Multivariate models

In what follows, we present a series of multivariate OLS regression models through which we can build a picture of the social and geographical bases of support for stem-cell research. The analytic sample for all of our models contains 10761 respondents. The reduction in sample size is principally due to the fact that not all questions were asked of all respondents in both of the surveys combined with some item non-response. The first set of models (1 to 3) assesses the association with approval of region and religiosity and the interaction between these two variables. A fourth model adds controls for age, gender and education to check the robustness of the associations observed in the first set. Two further sets of models (5–8) include moral (sanctity of life ethic) and benefit (utilitarian ethic) attitudes and, again, the interaction between these factors and region. In this way, we can assess the extent to which both moral and benefit based concerns are implicated in approval as well as the relative importance of these in determining levels of approval in the U.S., Canada and Europe. The final model (9) combines all of these variables.

Table 3 presents unstandardized regression coefficients for each of these models. In model 1, dummy variables for Canada and the U.S. have positive coefficients, which confirm what we saw in the bivariate tables presented earlier: namely, that levels of approval are higher in North America than in Europe. In model 2 we added religiosity and confirm, again, that people for whom religion is more important are less approving of stem-cell research, irrespective of the continent in which they live. Our first hypothesis was that religion would be more important in understanding attitudes to stem-cell research in the U.S. than elsewhere. In model 3 we included product-term interactions of religiosity and region and find that the negative association of religiosity with approval in the U.S. is indeed stronger than in Canada and Europe. The statistically significant coefficient for this interaction is -0.08, which, when combined with the main effect for religiosity of -0.11, means that each unit increase in the religiosity scale is associated with a decline in approval of around 0.2 for U.S. citizens. This relationship is robust when we control for age, gender and education in model 5, with little change in the coefficients denoting region.

**Table 3:** OLS regression models predicting approval of stem-cell research (unstandardized estimates and t-values).

	(1)	(2)	(3)	(4)	(5)	(6)	(7)	(8)	(9)
U.S.	0.34***	0.41***	0.45***	0.44***	0.32***	0.22***	0.43***	0.44***	0.30***
	(13.17)	(16.19)	(16.32)	(15.83)	(11.84)	(7.86)	(18.38)	(18.74)	(12.00)
CAN	0.36***	0.37***	0.37***	0.36***	0.26***	0.22***	0.40***	0.39***	0.29***
	(13.16)	(13.76)	(13.69)	(13.44)	(9.89)	(7.97)	(17.04)	(16.97)	(11.52)
Religiosity		-0.12***	-0.11***	-0.10***	-0.07***	-0.07***	-0.05***	-0.05***	-0.03***
		(-16.82)	(-13.52)	(-11.84)	(-8.61)	(-9.25)	(-7.53)	(-6.93)	(-5.13)
Relig x U.S.			-0.08***	-0.09***	-0.05*	0.02	-0.02	-0.04*	-0.00
			(-3.54)	(-4.05)	(-2.14)	(0.95)	(-1.06)	(-2.33)	(-0.16)
Relig x CAN			-0.03	-0.03	-0.02	0.00	0.00	-0.03	-0.02
			(-1.09)	(-1.37)	(-0.81)	(0.15)	(0.07)	(-1.43)	(-0.83)
Female				-0.06***	-0.04**	-0.05**	-0.05***	-0.04***	-0.03**
				(-3.69)	(-2.67)	(-3.05)	(-3.64)	(-3.37)	(-2.67)
25–34				0.02	0.01	0.01	-0.00	0.00	-0.00
				(0.50)	(0.42)	(0.37)	(-0.02)	(0.02)	(-0.00)
35–44				0.05	0.03	0.03	0.02	0.02	0.01
				(1.51)	(0.95)	(0.85)	(0.67)	(0.77)	(0.38)
45–54				0.02	0.00	0.00	-0.02	-0.02	-0.03
				(0.64)	(0.04)	(0.00)	(-0.55)	(-0.49)	(-0.89)
55–64				-0.01	-0.05	-0.05	-0.06	-0.06	-0.08**
				(-0.22)	(-1.27)	(-1.38)	(-1.80)	(-1.90)	(-2.71)
65+				-0.01	-0.04	-0.04	-0.04	-0.04	-0.06
				(-0.15)	(-1.12)	(-1.16)	(-1.13)	(-1.20)	(-1.92)
High school				0.07**	0.06**	0.07**	0.02	0.02	0.01
				(3.12)	(2.74)	(2.92)	(1.14)	(0.87)	(0.75)
College+				0.23***	0.18***	0.18***	0.10***	0.09***	0.07***
				(9.15)	(7.72)	(7.77)	(4.81)	(4.44)	(3.63)
Still studying				0.22***	0.18***	0.18***	0.09**	0.08*	0.07
				(5.27)	(4.47)	(4.52)	(2.60)	(2.34)	(1.87)
EducationDK				0.04	0.02	0.02	0.00	-0.00	-0.01
				(0.52)	(0.29)	(0.31)	(0.04)	(-0.00)	(-0.14)
Moral					0.24***	0.20***			0.13***
					(30.35)	(22.46)			(16.29)
Moral x U.S.						0.25***			0.17***
						(10.11)			(7.01)
Moral x CAN						0.14***			0.12***
						(5.19)			(4.39)
Benefit							0.56***	0.62***	0.59***
							(63.07)	(59.06)	(57.09)
Ben x U.S.								-0.16***	-0.31***
								(-6.56)	(-11.33)
Ben x CAN								-0.25***	-0.35***
								(-8.90)	(-11.43)
*R*^2^	0.028	0.053	0.038	0.056	0.136	0.144	0.316	0.324	0.356

*t* statistics in parentheses. N = 10761

* p < 0.05

** p < 0.01

*** p < 0.001

Looking across Table 3 to model 6, we introduce our measure of moral judgment on stem-cell research. The effect is statistically significant and positive–in other words, the belief that stem-cell research is morally acceptable is associated with its approval. More interesting is that when we examine the interaction between region and moral attitude, we find a significant positive effect for both Canada and the U.S. That is to say, moral attitudes are more consequential for public approval in both North American countries compared to Europe.

In models 7 to 9 we tested the same idea, only in this case with beliefs about the potential benefits of stem-cell research. The coefficient for benefit (0.56) in model 7 is substantial and statistically significant. Approval of stem-cell research is strongly tied to beliefs about its potential benefits. To see whether these beliefs are more or less important in different regions, in model 8 we added the interaction terms as we did for moral attitudes. We find that they are negative for both Canada and the U.S: the effect of benefit beliefs on approval is smaller in North America than in Europe. That is to say that how useful North Americans believe stem-cell research to be is less important than it is for Europeans in determining how likely they are to approve of it. In the final model, we simultaneously fitted both interactions. As can be seen, the results are substantively unchanged, with all the interaction terms remaining significant and of similar magnitude. Important also to note is that our final model explained more than 30 percent of the variation in approval for stem-cell research. While this leaves open the possibility that other systematic unmeasured factors are important for a full understanding of public opinion on the issue, it does mean that the dimensions we have investigated here are of substantial importance.

### Robustness check

In treating 25 European countries a single bloc, we of course run the risk of masking substantial heterogeneity. To check whether this was a significant problem, we re-estimated the models within each of the 25 countries separately. We found that the pattern of coefficients was very similar within each European country to the overall results treating countries as a bloc, as reported above. In particular, no European country’s ranking in the size of coefficients for moral and benefit resemble those for the U.S. and Canada. We are confident, therefore, that we have identified dimensions of difference that are substantially ‘transatlantic’ in nature.

## Discussion

Despite elite voices that have been critical of stem-cell research in Europe, Canada and the U.S., the majority of the public has been supportive for the past decade. But the fault lines of contemporary bioethics are reflected in public views. Religion plays an important part in many people’s lives, and appeals to religion have been prominent in the stem-cell debate, especially in the U.S. but also in some some predominantly Catholic European countries [34]. Yet in the case of stem-cell research it does not always result in positions of closure, pitting science against religion or religious versus secular world-views. While a sizeable fraction of the most religious want a veto on stem-cell research, many equally religious people have been willing to support it.

Our findings are consistent with the premise that public views on stem-cell research are framed by at least two key dimensions–moral concerns and beliefs about benefits. Although it would be unwise from survey data such as this to conclude that publics are weighing formal ethical positions against each other, we believe that the attitudinal positions we identify in our analyses to some degree map on to conventional ethical positions towards human life: namely, the sanctity of life and the quality of life. Even taking into account standard demographic groupings, perceptions of the benefits and of the moral acceptability of stem-cell research point to differences in views that go beyond what one might expect based on, for instance, religious and educational cleavages in the population. That is to say, both the more and less religious can differ in how much these two ethical dimensions underpin their opinions about stem-cell research. Moreover, the relative importance of these ethical positions differs not only between individual citizens but also between the U.S., Canada and Europe. In Europe particularly, given the greater relative importance of perceived benefits of stem-cell research, public support is likely to be strongly conditional on perceived progress towards the promised cures for diseases. In the U.S., where considerations of moral acceptability assume more importance than consideration of benefits, continued support for stem-cell research may be conditional to a greater extent on how embryonic stem-cells are obtained. whether political debate develops in a direction that emphasises sanctity of life over other considerations. At all events, the benefits need to be perceived as greater by Americans compared Europeans in order to be persuasive in the face of strong moral concerns.

Finally, while support for stem-cell research has grown in the U.S. during the past decade, there are political currents that exist–for instance the partisan positions on abortion–that may yet lead to a reversal of the trend. Some commentators have suggested that we have entered a ‘post-truth’ era [35]. If this is really so, we can expect to see shared values and norms, or indeed ‘public ethics’, becoming even more relevant for understanding how sensitive technologies enter into the public sphere and how public policy is framed in response.

## Supporting information

S1 File analysis stemcellplosone.do. Stata do-file containing code used to run models.
(DO) Click here for additional data file.

S2 File stemcell sensitivity analysis.xlsx. Excel file containing results of robustness check.
(XLSX)

S3 File stemcelldata.dta. Data file in Stata format used in the analysis.
(DTA)
